# Cryptoccose neuroméningée chez un patient VIH négatif à Bamako, Mali

**DOI:** 10.48327/mtsi.v4i4.2024.593

**Published:** 2024-01-11

**Authors:** Farimadiané COULIBALY, Yama DOUMBIA, Hama Hamidou ISSA, Sounkalo DAO

**Affiliations:** 1 Service des maladies infectieuses et tropicales du Centre hospitalier universitaire du Point G, Bamako, Mali; 2 Faculté de médecine et d'odontostomatologie de l’Université des sciences, des techniques et des technologies de Bamako, Mali; 3 Centre universitaire clinique et de recherche (UCRC), Bamako, Mali

**Keywords:** Cryptococcose neuroméningée, VIH négatif, Bamako, Mali, Afrique subsaharienne, Neuromeningeal cryptococcosis, HIV-negative, Bamako, Mali, Sub-Saharan Africa

## Abstract

**Introduction/Justification:**

La cryptococcose neuroméningée (CNM) est une infection fongique très fréquente chez les patients immunodéprimés par une infection à VIH. Sa rareté en dehors du VIH/sida doit faire rechercher systématiquement d'autres facteurs favorisant une immunodépression et nécessite de réaliser un diagnostic différentiel. Nous rapportons un cas de CNM chez un patient immunocompétent, hospitalisé au CHU du Point G de Bamako au Mali.

**Description du cas:**

Le patient présentait une fièvre (39,3 °C), une altération de l’état général, un score de Glasgow à 10/15 sans déficit moteur, une raideur de la nuque et des céphalées en casque rebelles aux antalgiques. Il avait un taux de CD4 à 932 cellules/mm^3^. Aucune autre pathologie immunodépressive n'a été retrouvée. Le diagnostic de CNM a été retenu devant des arguments cliniques et microbiologiques. Le patient a été traité avec succès à Bamako par un protocole alternatif à base de fluconazole, traitement plus accessible, moins couteux avec moins d'effets secondaires que l'amphotéricine B.

**Discussion/conclusion:**

La survenue d'une cryptococcose sur un terrain immunocompétent est rare. Elle peut cependant survenir sans facteur favorisant. Le fluconazole est une alternative thérapeutique efficace.

## Abstract (en)

**Introduction/Rationale:**

Neuromeningeal cryptococcosis (NMC) is a common fungal infection in immunocompromised patients with HIV infection. Its rarity outside HIV/AIDS requires a systematic search for other immunosuppressive factors and a differential diagnosis. We report a case of NMC in an immunocompetent patient admitted to the Point G University Hospital Center in Bamako, Mali.

**Case description:**

The patient presented with fever (39.3°C), general deterioration, Glasgow Coma Scale score of 10/15 without motor deficits, neck stiffness, and helmet-like headache unresponsive to analgesics. The patient had a CD4 count of 932 cells/mm^3^. No other immunosuppressive conditions were noted. The diagnosis of NMC was confirmed based on clinical and microbiologic findings. The patient was successfully treated in Bamako using an alternative protocol with fluconazole, a more accessible, less expensive treatment with fewer side effects compared to amphotericin B.

**Discussion/Conclusion:**

The occurrence of cryptococcosis in an immunocompetent host is rare. However, it can occur in the absence of predisposing factors. Fluconazole is an effective alternative therapy.

## Introduction

La cryptococcose neuroméningée (CNM) est une infection opportuniste fongique due à *Cryptococcus* spp. Elle constitue la première cause de méningite fongique et la troisième cause de méningite chronique [2]. Rare chez sujet VIH négatif, elle survient souvent au cours du VIH/sida lorsque le taux de T CD4 est inférieur à 100 cellules/mm^3^ [3, 5].

Nous rapportons un cas de CNM chez un patient immunocompétent dont le taux des lymphocytes T CD4 était normal.

## Cas clinique

Il s'agit d'un patient de 31 ans, commerçant de tissus, originaire de Bamako, admis dans le service des maladies infectieuses et tropicales du CHU du Point G (Bamako, Mali) le 16 août 2023 pour une altération de la conscience, des céphalées en casque et une fièvre. La symptomatologie s'est installée assez rapidement en trois semaines et le patient a été initialement traité dans une clinique privée pour un paludisme simple, confirmé par une goutte épaisse, à base d'artéméther-luméfantrine et de paracétamol par voie orale, entrainant une légère amélioration. Le patient n'a aucun antécédent médico-chirurgical connu, il n'est pas alcoolo-tabagique, il n'utilise pas de dermocorticoïdes ni de thérapie immunosuppressive et n'a aucune notion de voyage. Cependant, il signale pratiquer l’élevage de pigeons à son domicile.

L'examen physique retrouve une fièvre (39,3 °C), une altération de l’état général, un trouble de la conscience avec un score de Glasgow à 10/15 sans déficit moteur, une raideur de la nuque et des céphalées en casque résistant aux antalgiques de palier I. L'examen pleuropulmonaire, de la peau et ophtalmologique est sans particularités.

**Figure 1:**
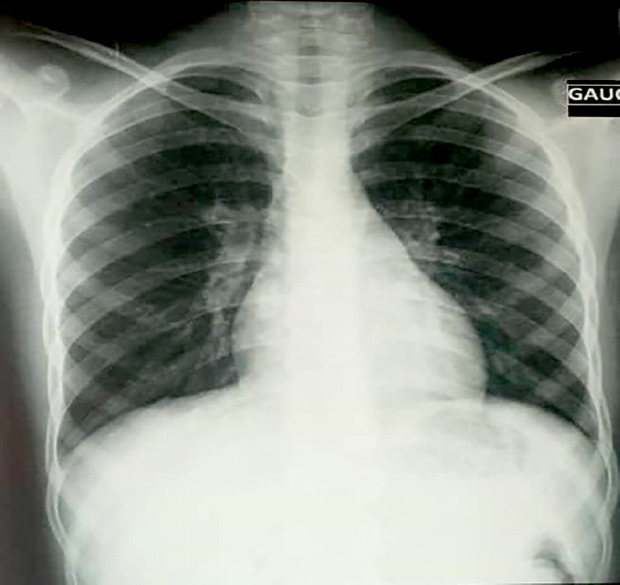
Radiographie du thorax de face : normale

**Figure 2:**
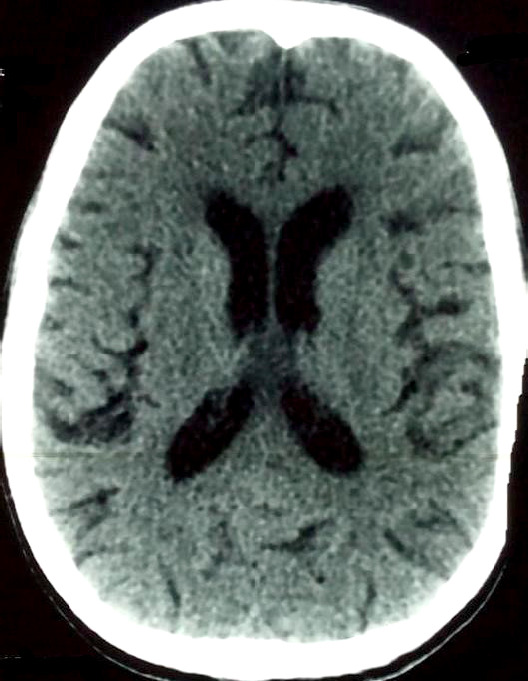
Tomodensitométrie cranio-encéphalique en coupe axiale avant injection de produit de contraste : normale

**Figure 3:**
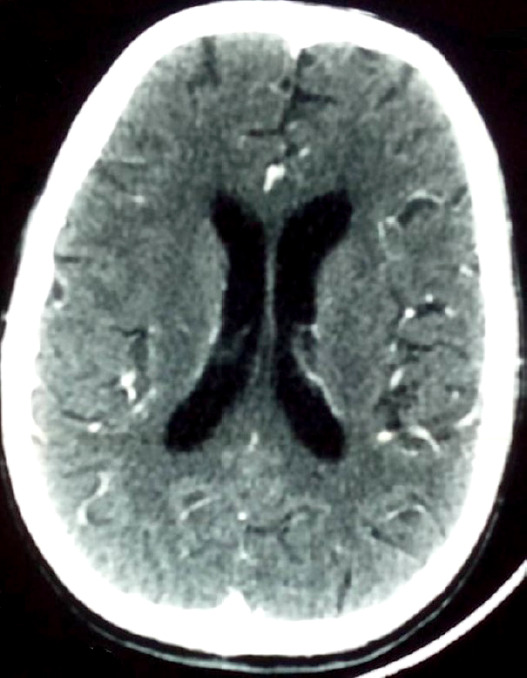
Tomodensitométrie cranio-encéphalique en coupe axiale après injection de produit de contraste : normale

La radiographie thoracique de face montre un aspect normal (Fig. 1). La tomodensitométrie cranio-encéphalique (TDM) réalisée en urgence avec (Fig. 2) et sans (Fig. 3) injection de produit de contraste révèle un aspect normal. La ponction lombaire (PL) montre un liquide d'aspect clair, eau de roche.

L'hémogramme retrouve une hyperleucocytose (14 700 GB/µl), à prédominance neutrophiles (10 237 PNN/µl). Le taux de lymphocytes T CD4 est de 932 cellules/mm^3^. La protidémie est normale (70,61 g/l). À la recherche d'autres maladies immunodépressives sont réalisées deux sérologies VIH qui sont négatives (en ELISA pour le dépistage et au Western blot pour la confirmation), deux glycémies à jeun qui sont normales (5,94 et 5,72 mmol/l) et une électrophorèse de l'hémoglobine qui est aussi normale (Hb-A à 96,8 %, Hb-A2 à 3,2 %). Les sérologies des hépatites virales (AgHBs, Ac anti-HBc, VHC) sont négatives. Le bilan rénal est normal (clairance de la créatininémie à 114,1 ml/min, protéinurie de 24 h à 0,04 g/l). Les marqueurs tumoraux sont normaux (alpha-fœtoprotéine à 4,5 ng/ml, antigène tumoral CA-125 à 30 U/ml, antigène tumoral CA-15-3 à 15 U/ml et antigène carcino-embryonnaire à 2,6 ng/ml). Ces résultats permettent d’écarter une infection à VIH, un diabète, une drépanocytose, les hépatites virales B et C, une insuffisance rénale et un cancer.

**Figure 4:**
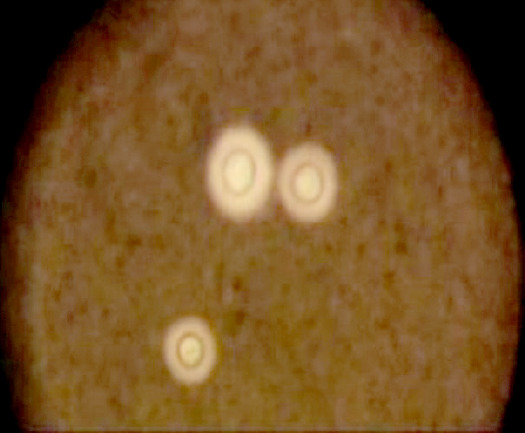
L'examen direct du LCS relève la présence de corps ovalaires entourés d'une auréole blanche après coloration à l'encre de Chine

**Figure 5:**
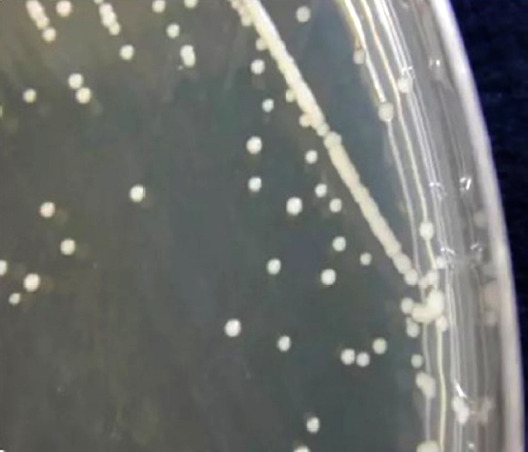
La culture du LCS sur milieu de Sabouraud après 48 heures d'incubation, relève des colonies crémeuses, à bords réguliers, brillantes, blanchâtres puis ocres

L'examen microbiologique du liquide cérébrospinal (LCS) objective une leucocytose à 55 éléments/mm^3^ (20 % de polynucléaires neutrophiles et 80 % de lymphocytes), une hyperprotéinorachie (0,95 g/l), une hypoglycorachie (0,50 g/l avec un rapport glycorachie/glycémie à 0,62). La chlorurorachie est de 120 mmol/l et la lactatorachie de 3,1 mmol/l. L'examen direct du LCS après coloration à l'encre de Chine relève la présence de corps ovalaires entourés d'une auréole blanche, très nette, régulière, de taille variable évoquant des levures encapsulées (Fig. 4). La culture sur milieu de Sabouraud après 48 heures d'incubation, révèle des colonies crémeuses, à bords réguliers, brillantes, blanchâtres, puis ocres (Fig. 5). Le test à l'uréase positif sur le milieu urée-indole, l'assimilation de l'inositol, la phénol-oxydase attestée par la coloration marron des colonies sur le milieu de Pal modifié, l'inositol assimilé et le glucose non fermenté sont en faveur de *Cryptococcus neoformans* réalisé au laboratoire *Malaria and Training Center* (MRTC) de Bamako (Mali). L'analyse bactériologique du LCS est normale, à l'examen direct comme à la culture. La détection d'antigènes circulants du cryptocoque dans le sang par la technique d'agglutination au latex est positive au 1/10 000.

Devant ces résultats, le diagnostic de méningite fongique à *Cryptococcus neoformans* est retenu. Le patient est traité par fluconazole à 1 200 mg/jour en trois perfusions pendant deux semaines (phase d'induction) puis 800 mg/jour *per os* pendant 10 semaines (phase de consolidation), puis 200 mg/jour *per os* (phase d'entretien) pour une durée de 12 mois, associé initialement aux antalgiques de palier II à base de tramadol 100 mg (une ampoule toutes les 8 heures en intraveineuse). Deux ponctions lombaires soustractives (100 ml/PL), à intervalle de 48h, sont réalisées.

L’évolution est favorable à 14 jours de traitement, marquée par l'apyrexie, la reprise de la conscience (score de Glasgow 15/15), l'amendement des céphalées, la disparition de la raideur de la nuque. Le contrôle du LCS avec coloration à l'encre de Chine est normal. À la fin de la phase de consolidation, le patient a une bonne tolérance et observance thérapeutique, et la culture du LCS sur milieu de Sabouraud est stérile. Il sort de l'hôpital 34 jours plus tard.

## Discussion

*Cryptococcus* spp. est une levure cosmopolite dont le tropisme pour le système nerveux central est marqué [6]. Dans la littérature, la prévalence de la CNM chez les PvVIH et les sujets VIH-négatifs est respectivement de 5,1 et 0,6 %, et *C. neoformans* est la principale espèce responsable [10]. La cryptococcose est relativement rare et survient le plus souvent sur un terrain de déficit profond de l'immunité cellulaire. Les spores sont présentes dans les sols, les débris végétaux et organiques (fientes de pigeons ou de chauve-souris).

La transmission par inhalation des spores peut être liée à certaines activités à risque dont celles entraînant un contact avec des oiseaux [4]. En l'absence d'antécédent de pathologies pouvant être des facteurs d'immunodépression, l’élevage de pigeons de notre patient pourrait être le facteur environnemental de risque. Ce mode de contamination lié aux oiseaux différentie *C. neoformans* de *C. gattii,* champignon aussi présent en milieu tropical, en particulier en Afrique subsaharienne, infectant des patients souvent immunocompétents et dont le facteur environnemental de risque serait l'exposition aux plantes comme l'eucalyptus.

Après avoir éliminé une éventuelle immunosuppression ou une méningo-encéphalite tuberculeuse, le diagnostic positif repose sur l'analyse du LCS. L'aspect cytochimique du LCS peut ne pas être franc, mais il montre typiquement une hyperlymphocytose associée à une hypoglycorachie souvent modérée et inconstante. La recherche du cryptocoque se fait dans le LCS à l'aide de techniques directes à l’état frais, après coloration à l'encre de Chine ou sur la mise en culture en milieu de Sabouraud qui, outre l'identification de la levure, permet de réaliser un antifongigramme. La détection d'antigènes capsulaires spécifiques est l'un des meilleurs tests diagnostiques rapides existants en mycologie, utilisé sur des liquides de prélèvements et peut être réalisée par différentes méthodes (agglutination au latex, ELISA, test immunochromatographique) [4].

L'identification moléculaire du sérotypage et du génotypage des souches est appliquée pour l'identification des levures dans un but épidémiologique. L'identification repose sur la spectrométrie de masse de type MALDI-TOF qui peut permettre l'identification précise au sein des deux complexes d'espèces [9]. Dans notre cas, la réalisation de ces tests était impossible pour manque de réactifs.

Dans la CNM, le scanner est le plus souvent d'aspect normal, mais peut donner des anomalies aspécifiques telles que des micro-abcès, un aspect de granulome inflammatoire, une inflammation des méninges qui, dans le contexte d'une infection subaiguë ou chronique du système nerveux central, constituent des éléments d'orientations solides [12].

En pays endémique comme le nôtre, le diagnostic différentiel de la CNM se fait avec les autres causes de méningites à liquide clair, notamment la méningite tuberculeuse. Le tableau clinique peut parfois évoquer les autres méningites bactériennes (à liquide clair, syphilitique), les méningites virales (ourlienne, herpétique, cytomégalovirus), etc. [5]. Dans la littérature, le traitement de la cryptococcose est une urgence. L'amphotéricine B (0,7-1,0 mg/kg/j) associée au 5-fluocytosine (100 mg/kg/j) représentent le traitement de référence des CNM à la phase d'induction [11]. En raison du coût élevé de l'amphotéricine B et de la non-disponibilité de la 5-fluocytosine au Mali, notre patient a bénéficié du fluconazole à forte dose, traitement proposé par plusieurs auteurs, en particulier en l'absence d'immunodépression [1, 7, 8].

L’évolution de la CNM est fatale en absence de traitement et la létalité reste élevée, y compris en cas de traitement bien conduit.

Les séquelles neurologiques les plus décrites sont les troubles cognitifs, l'hypoacousie, l'hydrocéphalie obstructive et la cécité qui peuvent être secondaire, soit à l'hypertension intracrânienne, soit à une invasion directe du nerf optique par les cryptocoques [11].

## Conclusion

La CNM est très fréquente chez les immunodéprimés au cours du sida. Elle peut cependant survenir chez des sujets sans facteur apparent d'immunodépression. Bien que rare chez les immunocompétents, la CNM doit être recherchée systématiquement en cas de suspicion de méningite lymphocytaire ou de méningo-encéphalite. Le fluconazole peut suppléer au manque d'amphotéricine B dans les pays où l'indisponibilité et le coût élevé de cet antimycosique de référence ne permettent pas de l'utiliser pour le traitement des méningo-encéphalites à cryptocoques.

## Consentement éclairé

Notre patient a donné son consentement éclairé pour la publication de son dossier médical sous anonymat.

## Remerciements

Nos remerciements s'adressent aux docteurs Mariame Soumaré, Dramane Sogoba, et Oumar Magassouba, ainsi qu'au professeur Yacouba Cissoko et Issa Konaté pour leurs contributions dans la prise en charge du patient et pour leurs apports critiques à cet article. Nous remercions également le professeur Safiatou Niaré Doumbo et à toute son équipe pour leurs apports dans le diagnostic mycologique chez ce patient.

## Contributions des auteurs

Farimadiané COULIBALY : Conception du cas clinique, prise en charge du patient, revue de la littérature, rédaction du manuscrit.

Hama Hamidou ISSA : Prise en charge du patient, revue de la littérature, apport critique, approbation de la version finale à publier.

Yama DOUMBIA, Sounkalo DAO : Prise en charge du patient, apport critique, correction du manuscrit et approbation de la version finale à publier.

## Conflits d'intérêts

Les auteurs ne déclarent aucun lien d'intérêts lié à ce travail.
