# Supplementary material for: Neuromeningeal cryptoccosis in an HIV-negative patient in Bamako, Mali
Source: Med Trop Sante Int. 2024 Jan 11;4(4):mtsi.v4i4.2024.593. [Article in French] doi: 10.48327/mtsi.v4i4.2024.593 (PMC11892387; doi:10.48327/mtsi.v4i4.2024.593)
Supplement: Related texts

# Research article: Neuromeningeal cryptoccosis in an HIV-negative patient in Bamako, Mali

## Introduction

Neuromeningeal cryptococcosis (NMC) is an opportunistic fungal infection caused by *Cryptococcus* spp. It is the most common cause of fungal meningitis and the third most common cause of chronic meningitis [2]. Rare in HIV-negative subjects, it often occurs during the course of HIV/AIDS when the CD4 T-cell count is below 100 cells/mm^3^ [3, 5].

We report a case of NMC in an immunocompetent patient with a normal CD4 T-cell count.

## Clinical case

A 31-year-old fabric merchant from Bamako was admitted to the Infectious and Tropical Diseases Department of CHU du Point G (Bamako, Mali) on August 16, 2023, with altered consciousness, headache, and fever. The symptoms developed rather rapidly over three weeks, and the patient was initially treated in a private clinic for uncomplicated malaria, confirmed by a thick droplet, with artemether-lumefantrine and oral paracetamol, with slight improvement. The patient has no known medical or surgical history, is not an alcoholic or a smoker, is not using dermocorticoids or immunosuppressive therapy, and has no plans to travel. However, he reports raising pigeons at home.

Physical examination reveals fever (39.3°C), altered general condition, altered consciousness with Glasgow score of 10/15 and no motor deficit, stiff neck, and headache refractory to grade I analgesics. Pleuropulmonary, skin, and ophthalmologic examinations were unremarkable.

The frontal chest x-ray was normal (Fig. 1). Emergency cranioencephalic computed tomography (CT) scan with (Fig. 2) and without (Fig. 3) contrast showed a normal appearance. Lumbar puncture (LP) revealed a clear, rock-water fluid.

**Figure 1:** Chest X-ray from the front: normal

**Figure 2:** Axial cross-sectional cranioencephalic computed tomography before contrast injection: normal

**Figure 8:** Axial cross-sectional cranioencephalic computed tomography after contrast injection: normal

The blood count showed hyperleukocytosis (14,700 GB/µL), predominantly neutrophilic (10,237 PNN/µL). The CD4 T-lymphocyte count was 932 cells/mm^3^. Protidemia was normal (70.61 g/L). Two negative HIV serologies (ELISA for screening and Western blot for confirmation), two normal fasting blood sugars (5.94 and 5.72 mmol/l) and a normal hemoglobin electrophoresis (Hb-A 96.8%, Hb-A2 3.2%) were performed to check for other immunosuppressive diseases. Viral hepatitis serologies (HBsAg, anti-HBc, HCV) were negative. Renal function tests were normal (creatinine clearance 114.1 mL/min, 24-hour proteinuria 0.04 g/L). Tumor markers were normal (alpha-fetoprotein 4.5 ng/mL, tumor antigen CA-125 30 U/mL, tumor antigen CA-15-3 15 U/ml and carcinoembryonic antigen 2.6 ng/mL). These results excluded HIV infection, diabetes, sickle cell disease, viral hepatitis B and C, renal failure and cancer.

Microbiological examination of the cerebrospinal fluid (CSF) revealed a leukocytosis of 55 elements/mm^3^ (20% neutrophils and 80% lymphocytes), hyperproteinorachia (0.95 g/L), hypoglycorachia (0.50 g/L with a glycorachia/glycemia ratio of 0.62). Chlororachia was 120 mmol/L and lactatorachia 3.1 mmol/L. Direct examination of the CSF after staining with India ink revealed oval bodies surrounded by a sharp, regular white aureole of variable size, suggestive of encapsulated yeasts (Fig. 4). Culture on Sabouraud medium after 48 hours’ incubation revealed creamy colonies with regular edges, shiny, whitish then ochre (Fig. 5). The positive urease test on urea-indole medium, the assimilation of inositol, the phenol-oxidase attested by the brown coloration of colonies on modified Pal medium, the assimilated inositol and unfermented glucose are in favor of *Cryptococcus neoformans* carried out at the Malaria and Training Center (MRTC) laboratory in Bamako (Mali). Bacteriological analysis of the CSF was normal, both on direct examination and culture. Detection of circulating cryptococcal antigens in blood by the latex agglutination technique is positive at 1/10,000.

**Figure 4:** Direct examination of the LCS reveals oval bodies surrounded by a white halo after tinting with China ink

**Figure 5:** LCS culture on Sabouraud medium after 48 hours of incubation shows creamy colonies, with regular edges, glossy, whitish and ochre

These results led to the diagnosis of *Cryptococcus neoformans* fungal meningitis.

The patient was treated with fluconazole 1,200 mg/day in three infusions for two weeks (induction phase), then 800 mg/day *per os* for 10 weeks (consolidation phase), then 200 mg/day *per os* (maintenance phase) for 12 months, initially in combination with tramadol 100 mg second step analgesics (one ampoule intravenously every 8 hours).

Two subtractive lumbar punctures (100 ml/PL) were performed at 48-hour intervals.

Progression was favorable after 14 days of treatment, with apyrexia, recovery of consciousness (Glasgow score 15/15), improvement of headache, and resolution of neck stiffness. Cerebrospinal fluid monitoring with India ink staining was normal. At the end of the consolidation phase, the patient had good tolerance and compliance, and the CSF culture on Sabouraud's medium was sterile. He was discharged 34 days later.

## Discussion

*Cryptococcus* spp. is a cosmopolitan yeast with marked tropism for the central nervous system [6]. In the literature, the prevalence of NMC in PHAs and HIV-negative individuals is 5.1% and 0.6%, respectively, with *C. neoformans* being the predominant species responsible [10]. Cryptococcosis is relatively rare and most commonly occurs in individuals with profound cellular immune deficiencies. Spores are found in soil, plants and organic debris (pigeon or bat droppings).

Transmission by inhalation of spores may be associated with certain high-risk activities, including contact with birds [4]. In the absence of a history of pathologies that could be factors in immunodepression, the pigeon farm of our patient could be the environmental risk factor. This bird-related mode of contamination distinguishes *C. neoformans* from *C. gattii,* a fungus that also occurs in tropical environments, particularly in sub-Saharan Africa, infecting patients who are often immunocompetent and whose environmental risk factor would be exposure to plants such as eucalyptus.

After exclusion of immunosuppression or tuberculous meningoencephalitis, a positive diagnosis is based on CSF analysis. The cytochemical appearance of the CSF may be ambiguous, but typically shows hyperlymphocytosis associated with hypoglycorachia, which is often moderate and inconsistent. Cryptococcus is detected in CSF by direct techniques in the fresh state, after staining with Indian ink, or by culture in Sabouraud's medium, which, in addition to identifying the yeast, allows an antifungiogram to be performed. Detection of specific capsular antigens is one of the best existing rapid diagnostic tests in mycology, used on sample fluids and can be performed by a variety of methods (latex agglutination, ELISA, immunochromatographic test) [4].

Molecular identification by serotyping and genotyping of strains is used to identify yeasts for epidemiologic purposes. Identification is based on MALDI-TOF mass spectrometry, which can provide accurate identification within both species’ complexes [9]. In our case, we were unable to perform these tests due to a lack of reagents. In NMC, the CT scan is usually normal in appearance, but may show aspecific abnormalities such as microabscesses, inflammatory granulomas, or meningeal inflammation, which are solid referral elements in the context of subacute or chronic central nervous system infection [12].

In endemic countries such as ours, the differential diagnosis of NMC is made with other causes of clear-fluid meningitis, in particular tuberculous meningitis. The clinical picture may also suggest other bacterial meningitis (clear-fluid, syphilitic meningitis), viral meningitis (mumps, herpetic, cytomegalovirus meningitis), etc. [5].

In the literature, treatment of cryptococcosis is considered an emergency. Amphotericin B (0.7-1.0 mg/kg/d) combined with 5-fluocytosine (100 mg/kg/d) is the standard treatment for NMC in the induction phase [11]. Due to the high cost of amphotericin B and the unavailability of 5-fluocytosine in Mali, our patient benefited from high-dose fluconazole, a treatment suggested by several authors, especially in the absence of immunosuppression [1, 7, 8].

The course of NMC is fatal without treatment, and the lethality rate remains high even with well-managed therapy.

The most described neurological sequelae are cognitive impairment, hypoacusis, obstructive hydrocephalus, and blindness, which may be secondary to either intracranial hypertension or direct cryptococcal invasion of the optic nerve [11].

## Conclusion

NMC is very common in immunocompromised patients with AIDS. However, it can also occur in subjects with no apparent immunosuppressive factors. Although rare in immunocompetent patients, NMC should be systematically investigated in cases of suspected lymphocytic meningitis or meningoencephalitis. Fluconazole can compensate for the lack of amphotericin B in countries where the unavailability and high cost of this reference antimycotic prevent its use in the treatment of NMC.

## Informed consent

Our patient gave his informed consent for the publication of his medical records anonymously.

## Acknowledgements

Our thanks go to Drs Mariame Soumaré, Dramane Sogoba, and Oumar Magassouba, as well as to Prof Yacouba Cissoko and Issa Konaté for their contributions in the care of the patient and for their critical contributions to this article. We would also like to thank Professor Safiatou Niaré Doumbo and her team for their contributions to the mycological diagnosis of this patient.

## Authors’ contributions

Farimadiané Coulibaly: Design of the clinical case, patient management, literature review, drafting of the manuscript.

Hama Hamidou ISSA: Patient management, literature review, critical input, approval of final version for publication.

Yama DOUMBIA, Sounkalo Dao: Patient management, critical input, correction of manuscript and approval of final version for publication.

## Conflicts of interest

The authors declare that they have no competing interests related to this work.
